# Structural Features Mediating Zinc Binding and Transfer in the AztABCD Zinc Transporter System

**DOI:** 10.3390/biom10081156

**Published:** 2020-08-06

**Authors:** Anusha Meni, Erik T. Yukl

**Affiliations:** Department of Chemistry and Biochemistry, New Mexico State University, Las Cruces, NM 88003, USA; meni@nmsu.edu

**Keywords:** zinc, ABC transporter, metal homeostasis, chaperone

## Abstract

Many bacteria require ATP binding cassette (ABC) transporters for the import of the essential metal zinc from limited environments. These systems rely on a periplasmic or cell-surface solute binding protein (SBP) to bind zinc with high affinity and specificity. AztABCD is one such zinc transport system recently identified in a large group of diverse bacterial species. In addition to a classical SBP (AztC), the operon also includes a periplasmic metallochaperone (AztD) shown to transfer zinc directly to AztC. Crystal structures of both proteins from *Paracoccus denitrificans* have been solved and suggest several structural features on each that may be important for zinc binding and transfer. Here we determine zinc binding affinity, dissociation kinetics, and transfer kinetics for several deletion mutants as well as a crystal structure for one of them. The results indicate specific roles for loop structures on AztC and an N-terminal motif on AztD in zinc binding and transfer. These data are consistent with a structural transfer model proposed previously and provide further mechanistic insight into the processes of zinc binding and transfer.

## 1. Introduction

Transition metals such as zinc, iron, manganese, and copper provide essential cellular functions, yet are highly toxic in their “free” forms or bound to incorrect targets. Thus, all living things must carefully maintain metal homeostasis. In general, this is accomplished by regulating the uptake and efflux of metals from the cell as well as by proteins that bind, store, and/or shuttle metals to their appropriate destinations. Proteins of the latter description are known as metallochaperones. Well-studied examples include the Atox1, CCS, and Cox17 metallochaperones that deliver copper to specific targets in eukaryotes [1], and the metallothioneins that buffer zinc concentration in eukaryotes [2] and a few bacteria [3]. A new family of intracellular zinc-binding GTPases called COG0523 has been identified in bacteria. These are often upregulated by zinc starvation [4,5,6] and may function as metallochaperones. However, direct evidence of metal transfer to target proteins has never been demonstrated, and their physiological functions are often unclear.

Metal binding proteins outside the cell are also important for bacterial metal homeostasis. ATP binding cassette (ABC) transporters are critical for high affinity uptake of transition metals and other nutrients. In addition to dimeric membrane permease and cytosolic ATPase proteins, bacterial ABC transporters rely on periplasmic (Gram-negative) or cell surface (Gram-positive) solute binding proteins (SBP) [7,8]. The SBP is essential to bind the substrate with high affinity and specificity and deliver it to the membrane permease for transport into the cell. SBPs have been categorized according to structure and substrate specificity [9], with members of group A-I mediating transport of zinc, manganese, or iron. The presence of a flexible loop rich in His/Asp/Glu residues near the metal binding site is indicative of zinc specificity [10]. These proteins have garnered considerable interest as potential antibiotic targets as they are required for high affinity zinc import and survival under the extremely zinc-limited conditions imposed by animal hosts [11,12].

The cluster A-I SBPs are not the only extracellular bacterial zinc metallochaperones. In a surprising number of cases, metal may be transferred to them by other metallochaperones. Examples of such chaperones include ZinT [13,14,15,16,17,18,19], the polyhistidine triad protein PhtD [20,21,22], and AztD [23]. Direct zinc transfer to an SBP has only been demonstrated for the latter two, and the mechanistic details of this process are unclear. This is typically the case despite the importance of zinc transfer processes in biology due to the lack of a convenient spectroscopic handle for this element. However, the kinetics of transfer from AztD to AztC can be easily followed by a roughly 2-fold increase in intrinsic protein fluorescence of AztC upon zinc binding [24]. This combined with crystal structures of both AztC [24,25] and AztD [26] make this system ideal for detailed mechanistic investigations of direct, protein-to-protein metal transfer as well as zinc binding/dissociation from solution.

**Figure 1:**
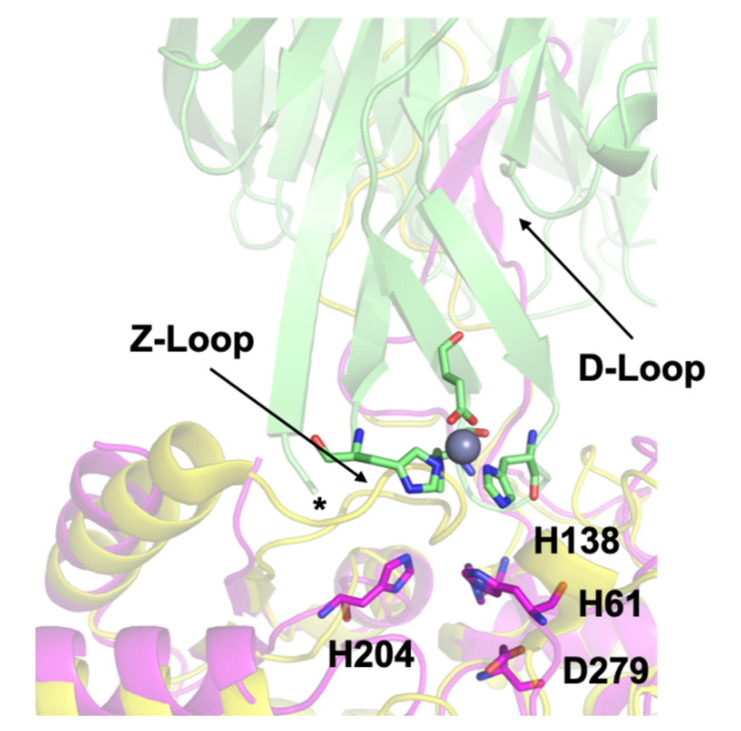
Docking model of the AztC-AztD transfer complex. AztD is shown in green with zinc binding residues shown as sticks colored according to element and zinc as a gray sphere. The first modeled N-terminal residue of AztD (His 28) is indicated by an asterisk. Apo AztC is shown in magenta with zinc binding residues labeled and shown as sticks colored according to element. Holo AztC superimposed on the apo form is shown in yellow, illustrating how the position of the Z-loop in this structure would block zinc transfer.

Our previous work culminated in a docking model showing the possible transfer complex formed between AztC and AztD (Figure 1). This model highlights several important structural features. First, the flexible loop of AztC that we refer to as the D-Loop projects into the central pore of the AztD beta-propeller structure. This loop and each of its 3 His residues are essential for transfer [25]. Secondly, AztC (residues 222–229) referred to as the Z-loop must be displaced away from the zinc binding site in order for the complex to form. This is true in the apo AztC structure, but this loop closes down over the zinc site in the holo structure. This was suggested to provide a means for dissociation of the complex after transfer. Finally, the 6 N-terminal residues after cleavage of the periplasmic targeting sequence of AztD (residues 22–27) are not observed in the crystal structure, but would be positioned at the interface with AztC according to the model. Residues 23–29 form a DHDHDHE motif that may bind zinc and/or facilitate transfer to AztC. Zinc binding to this N-terminal motif (NTM) was suggested by the observation of a third, relatively low-affinity binding site in addition to the two high-affinity sites in AztD [23].

Here we utilize deletion mutants and the convenient fluorescence behavior of AztC to characterize the role of each of the above structural elements on zinc dissociation from AztC and transfer from AztD. Each appears to have a unique effect on either or both processes that is consistent with the proposed model. Further, we sought to generate a mutant of AztC that would be capable of binding to AztD but unable to accept zinc from it. We reasoned that such a mutant would act as a competitive inhibitor of zinc transfer and would provide a means for determining the affinity between the two proteins. Thus, we generated an AztC mutant lacking the two zinc ligands His 138 and His 204 (Figure 1) that we refer to as H138/204A, and we evaluated its ability to competitively inhibit transfer to WT AztC. Remarkably, the presence of this mutant actually enhanced the rate of transfer from AztD. This last intriguing finding suggests additional complexity to the transfer event and provides avenues for further study of this important process.

## 2. Materials and Methods

### 2.1. Expression and Purification of Proteins

WT and mutant AztC [24] and AztD [23] were expressed and purified as previously described. All mutants were generated using the Q5^®^ Site-Directed Mutagenesis Kit (New England BioLabs, Ipswich, MA, USA) and confirmed by plasmid sequencing. Presence and purity of all proteins were confirmed using SDS-PAGE, and concentration was measured using extinction coefficients determined by the method of Edelhoch [27]. Apo proteins were generated as previously by dialysis at 4 °C against 50 mM NaOAc buffer pH 4.5, 50 mM EDTA, and 150 mM NaCl followed by dialysis against 50 mM tris buffer pH 8.0, 150 mM NaCl, and 3.4 g/L Chelex^®^ (Bio-Rad, Hercules, CA, USA).

### 2.2. Mag-Fura 2 Competition Assay

Zinc binding affinities were measured using a Mag-Fura 2 (MF2, Invitrogen, Carlsbad, CA, USA) competition assay derived from Golynskiy et al. [28] as previously described [23,24]. All fluorescence measurements were made using a Varian Cary Eclipse (Palo Alto, CA, USA) fluorescence spectrophotometer with entrance and exit slits set to 10 nm. Protein concentration was measured before each experiment and MF2 concentration was determined using an extinction coefficient at 369 nm of 22,000 M^−1^ × cm^−1^ [28]. In each experiment, 1.0 μM apo protein and 0.5 μM MF2 in binding buffer (20 mM HEPES pH 7.4, 200 mM NaCl, 5% *v*/*v* glycerol treated with Chelex^®^ resin) were titrated with increasing concentrations of ZnCl_2_, keeping the total volume of titrant added to less than 10% *v*/*v*. Fluorescence excitation spectra were scanned from 250–450 nm while monitoring emission at 505 nm. Experiments were performed in triplicate and the fluorescence intensities at λ_ex_ = 330 nm were fit using the program DYNAFIT v. 4.05.103 [29,30] using scripts adapted from Golynskiy et al. [28]. Prior to each series of experiments, the affinity of MF2 for zinc in our buffer system was determined using DYNAFIT and used in our calculation of protein binding affinity.

### 2.3. Zinc Dissociation

Apo WT or mutant AztC was diluted to 1 µM in binding buffer in a stirred cuvette at ambient temperature. Protein was titrated with up to 3 equivalents of ZnCl_2_ and the fluorescence intensity monitored to ensure saturation. Dissociation was initiated by the addition of EDTA to 1.0 mM and the decay in emission intensity at 315 nm was monitored. Data was fit by a single exponential function using DYNAFIT [29,30].

### 2.4. Zinc Binding and Transfer by Intrinsic Fluorescence

All fluorescence measurements were made using a Varian Cary Eclipse fluorescence spectrophotometer as previously described. Titration experiments were performed with AztC proteins at 10 µM in binding buffer. These were titrated with ZnCl_2_ or AztD reconstituted with 2 equivalents of ZnCl_2_ followed by desalting into binding buffer using Zeba™ spin desalting columns (Pierce Biotechnology™, Rockford, IL, USA). In titrations of apo-AztC with holo-AztD, 5–15 min of equilibration was allowed between measurements. After addition of holo-AztD to 14 µM, 20 µM of ZnCl_2_ was added to determine AztC saturation.

Kinetic experiments were performed as previously described [26] with minor modifications. Apo AztC at 0.5 μM in binding buffer containing 1 mM EDTA was placed in a stirred cell at ambient temperature. Transfer was initiated by addition of varying concentrations of AztD reconstituted as above but omitting the desalting step. Fluorescence emission at 315 nm (λ_exc_ = 278 nm) was recorded over time. Slit widths were varied to optimize signal to noise and avoid saturation at high protein concentration. First order fits to the data were generated using the onboard Cary Eclipse software. Inhibition experiments were performed similarly to those above but included varying concentrations of H138/204A or ΔD-loop AztC in addition to WT AztC at 0.5 μM. Transfer was initiated by addition of 0.5 μM reconstituted WT AztD and monitored as above.

### 2.5. Crystallization and Structure Determination

ΔZ-loop AztC were crystallized under the same conditions used for holo WT [24]. Crystals were cryoprotected with mother liquor containing 10% glycerol prior to cryocooling in liquid nitrogen. Diffraction data was collected at 100 K on beamline 5.0.2 at the Advanced Light Source at Berkeley National Laboratory and indexed, integrated, and scaled with XDS v. 0.92 [31,32]. The WT AztC structure (PDB ID: 5W57) [24] was used as the search model for molecular replacement using Phaser-MR [33] module of Phenix v. 1.14-3260. Manual model building was done in Coot v. 0.7 [34] and further rounds refinement were done using Phenix Refine [35]. Coordinates of ΔZ-loop AztC have been deposited in the PDB with entry code 6XPN. Figures were prepared using Pymol v. 2.1, which was also used for pairwise structural alignments.

## 3. Results

### 3.1. Zinc Binding and Dissociation

**Figure 2:**
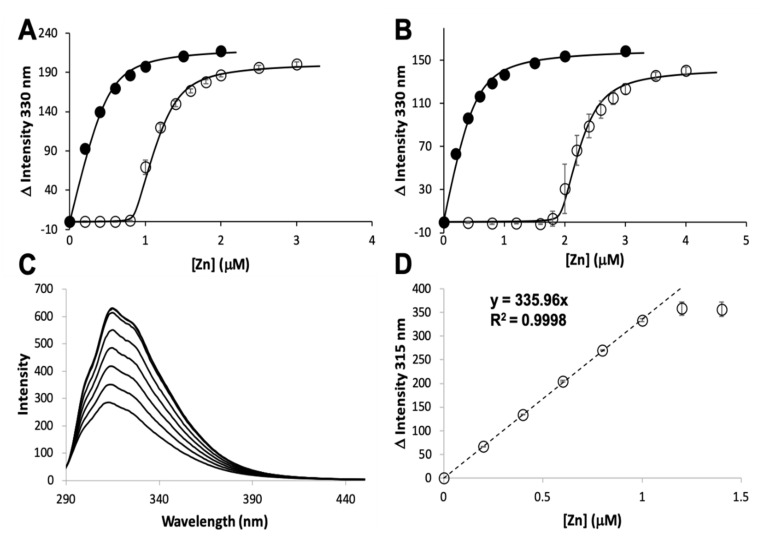
Zinc binding by AztC and AztD mutants. Intensity change of MF2 at 330 nm with increasing zinc in the absence (solid circles) and presence (empty circles) of apo H138/204A AztC (**A**) and ΔN-terminal motif (NTM) AztD (**B**). (**C**) Fluorescence emission spectra of H138/204A AztC (λ_exc_ = 278 nm) and (**D**) magnitude of the fluorescence intensity change with increasing zinc concentration. Titrations containing protein were performed in triplicate and error bars represent the standard deviation between measurements. Least squared fits are shown as solid lines for (**A**) and (**B**) and a dotted line for the linear fit in (**D**) with slope and R^2^ values indicated.

The zinc binding affinity of WT AztD, WT AztC, ΔD-Loop AztC, and ΔZ-Loop AztC were previously determined by competition assay with the fluorophore MagFura-2 (MF2) (Table 1). Here we apply this technique to H138/204A AztC and a deletion of the N-terminal motif (residues 23–29) in AztD (ΔNTM AztD) (Figure 2). The former binds approximately one equivalent (0.85) of zinc with a Kd below the detection limit of this assay (≤0.1 nM), making it indistinguishable from WT. ΔNTM AztD binds two equivalents of zinc with very high affinity. Previous work with WT AztD indicated some negative cooperativity between high affinity binding sites. Intriguingly, this appears to be largely eliminated in the ΔNTM mutant. Further, the third binding site in WT AztD is not observed, consistent with relatively low affinity zinc binding by this motif. We also evaluated zinc binding to H138/204A by intrinsic protein fluorescence (Figure 2C,D). Like WT, this mutant exhibits a roughly 2-fold increase in fluorescence emission intensity saturating at one equivalent of added zinc consistent with high affinity binding.

**Figure 3:**
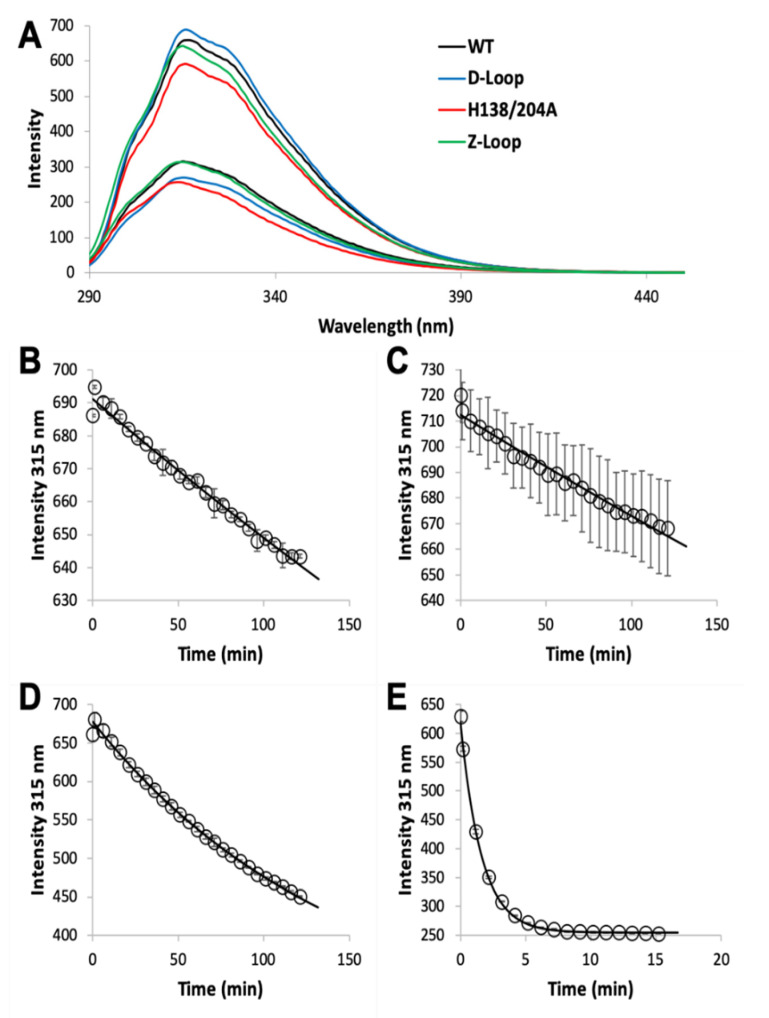
Zinc dissociation from WT and mutant AztC. (**A**) Fluorescence emission spectra of WT (black), ΔD-Loop (blue), ΔZ-Loop (green) and H138/204A (red) AztC before and after addition of saturating ZnCl_2_. Upon addition of excess EDTA, the emission intensity at 315 nm was tracked over time for WT (**B**), ΔD-Loop (**C**), ΔZ-Loop (**D**), and H138/204A (**E**) AztC. Dissociation experiments were performed in triplicate and error bars represent the standard deviation between measurements.

The MF2 assay does not reveal any significant differences between the affinities of the various AztC mutants, likely because each Kd is near or below the detection limit. Thus, we developed an intrinsic fluorescence assay to monitor the zinc off-rate for each (Table 1). Briefly, protein is saturated with zinc as determined by fluorescence emission (Figure 3A). Each of the AztC proteins investigated here is similar in terms of the magnitude of fluorescence intensity change upon zinc binding. Next, a large excess of EDTA is added to capture metal as it dissociates, and the decrease in fluorescence intensity is monitored over time as a direct measure of zinc loss. As expected, the zinc off-rates for WT and ΔD-Loop AztC are comparable and extremely slow (Figure 3B,C). However, deletion of the Z-loop causes a more than 5-fold increase in off-rate (Figure 3D), consistent with the function of this feature in closing down over the zinc site. Mutation of the two zinc ligands His138 and His204 causes a dramatic increase in off-rate, approximately 600 times faster than WT (Figure 3E). Assuming an upper limit to the Kd of 0.1 nM as indicated from the MF2 assay, this suggests that zinc binding to this mutant is near the diffusion limit, around 10^8^ M^−1^ × s^−1^.

### 3.2. Zinc Transfer from AztD

**Figure 4:**
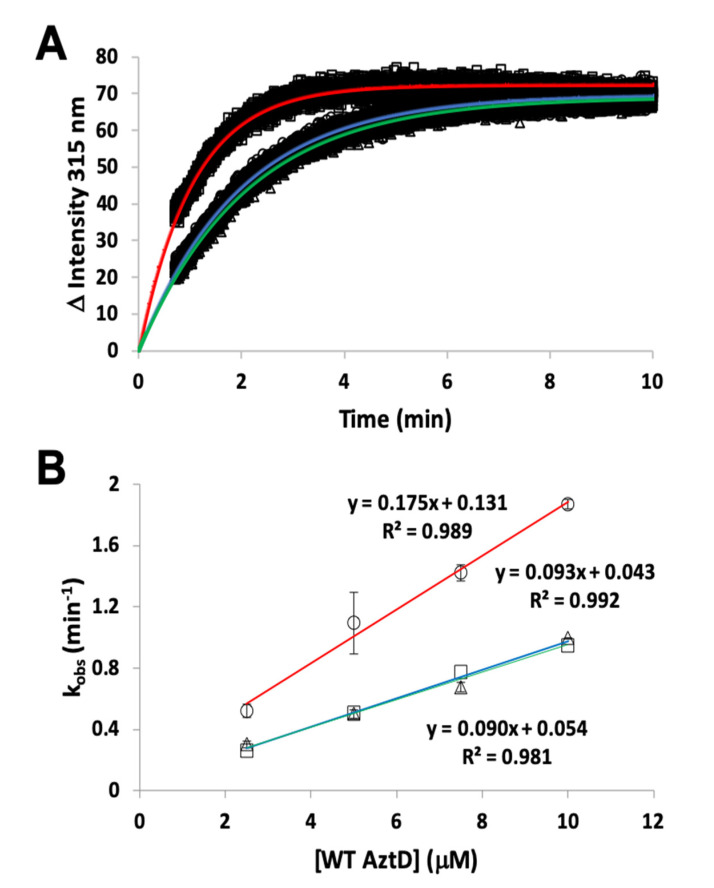
Kinetics of zinc transfer between mutants of AztD and AztC. (**A**) Representative kinetic data from standard fluorescence emission experiments for WT AztD to WT AztC (circles), ΔNTM AztD to WT AztC (triangles), and WT AztD to ΔZ-Loop (squares) with fits to a first order kinetic scheme (blue, green and red, respectively). (**B**) Observed first order rate constant versus AztD concentration for standard fluorescence data under pseudo-first order conditions. Error bars represent standard deviations from duplicate experiments (n = 2) except for the WT AztD to WT AztC experiment, which was run a single time to confirm previous data under current conditions. Symbols and linear fit colors are as for (**A**).

Transfer of zinc from AztD to AztC can be followed by the increase in AztC fluorescence upon zinc binding [23,25,26]. Previous work indicates that this process requires the presence of the D-Loop, but transfer was still observed to ΔZ-Loop AztC. However, the kinetics were not previously evaluated. Here we demonstrate that the rate of transfer from WT AztD to ΔZ-Loop AztC is increased approximately two-fold relative to WT AztC under the same conditions (Table 1, Figure 4). In contrast, deletion of the NTM from AztD appears to have no effect on the rate of transfer to WT AztC.

**Figure 5:**
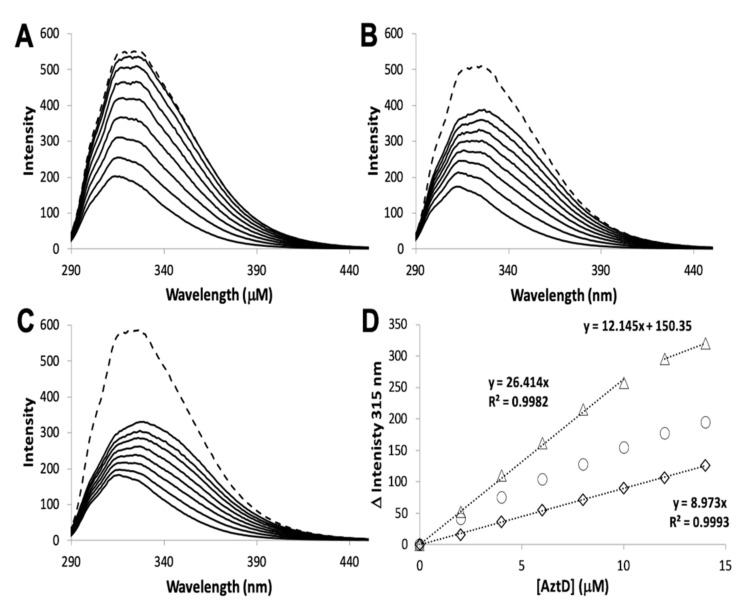
Zinc transfer by intrinsic fluorescence. Reconstituted WT AztD was titrated into apo WT (**A**), H138/204A (**B**), or ΔD-Loop (**C**) AztC. Fluorescence emission spectra (λ_exc_ = 278 nm) were recorded after each addition of AztD and the intensity change at 315 nm plotted as a function of AztD concentration (**D**) with equations for linear fits indicated where appropriate. A saturating concentration of ZnCl_2_ (1.1 mM) was added after the titration to assess whether transfer from AztD was complete (dotted line, A, B, and C).

The rate of transfer to H138/204A AztC could not be evaluated because of the rapid off-rate of zinc for this mutant. To determine if zinc transfer to this mutant from AztD was possible, it was titrated with holo AztD in the absence of EDTA (Figure 5). Although the fluorescence changes were smaller than for WT, they were nevertheless greater than for ΔD-Loop AztC. These changes also did not reach saturation after 1.4 equivalents of AztD were added and appear to have a roughly hyperbolic rather than linear dependence on AztD concentration. Cumulatively, these data suggest that zinc can be transferred to H138/204A AztC, but the rapid off-rate from this mutant allows an equilibrium to be established between zinc binding to it and AztD. This is in contrast to the virtually unidirectional transfer of zinc to WT AztC, which serves as a kinetic trap for zinc. This also differs from the behavior of ΔD-Loop AztC, which cannot access zinc from AztD at all.

### 3.3. Crystal Structure of ΔZ-Loop

**Figure 6:**
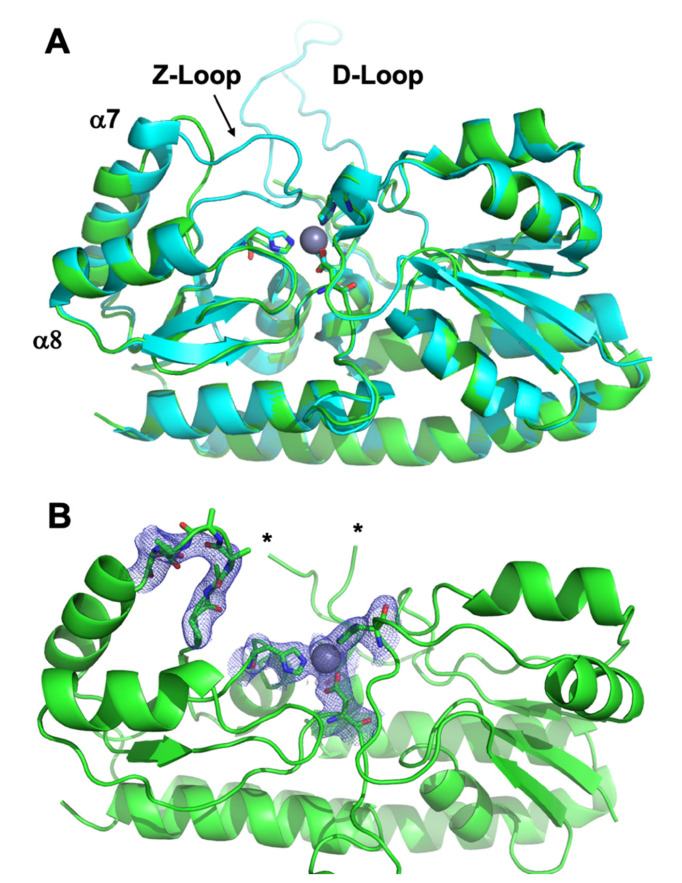
Crystal structure of ΔZ-Loop AztC. (**A**) Overlay of ΔZ-Loop (green) and WT (blue) AztC. Zinc binding residues are shown as sticks colored according to element and zinc is shown as a gray sphere. (**B**) ΔZ-Loop AztC showing 2Fo–Fc electron density contoured at 1.0σ for the truncated loop and zinc binding site. Asterisks indicate residues 116 and 134 between which residues could not be modeled due to a lack of electron density.

In order to determine what structural features of ΔZ-Loop AztC might mediate its more rapid transfer kinetics, a crystal structure was determined for this mutant (Table 2, Figure 6). The overall structure is very similar to that of the holo WT, with rmsd = 0.26–0.30 Å across all atoms depending on which chains are aligned. The zinc binding site is also completely superimposable. Residues 117–133 (the D-Loop) could not be modeled into either chain of the ΔZ-Loop AztC structure due to a lack of electron density. Residues 222–229 (the Z-Loop) are absent, leaving a run of four Ala residues. Electron density in this region is also somewhat weak, indicating some flexibility. The deletion shortens the loop between helices α7 and α8, preventing closure of the loop over the zinc binding site as is observed for the WT. The lack of any other significant structure changes as a result of the deletion suggest that this is the primary reason for the increase in zinc dissociation rate and transfer rate from AztD.

### 3.4. Competition Between AztC Mutants

**Figure 7:**
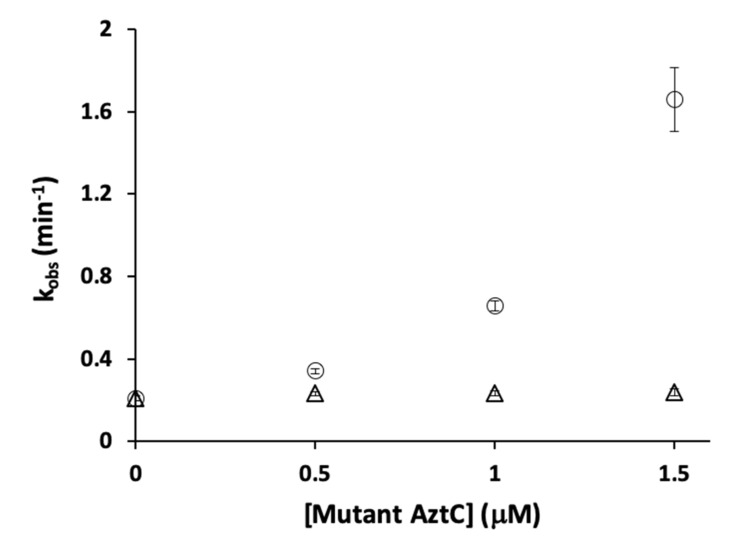
Kinetics of zinc transfer from WT AztD to WT AztC in the presence of mutant AztC. Observed first order rate constant versus H138/204A (circles) or ΔD-Loop (triangles) AztC concentration. WT AztC and AztD were both present at 0.5 μM, EDTA was present at 1 mM. Error bars represent standard deviations from triplicate experiments (n = 3).

We next investigated whether H138/204A AztC might act as a competitive inhibitor for the interaction between WT AztC and AztD. We reasoned that increasing concentrations of H138/204A AztC would slow the rate of transfer to WT AztC as this mutant can interact with AztD but would not show net transfer due to its rapid off-rate. In contrast, we actually observed a dramatic increase in the rate of transfer to WT AztC (Figure 7). To confirm that this was not a result of molecular crowding, we repeated the experiment with increasing concentrations of ΔD-Loop AztC, which had no impact on transfer to WT AztC.

## 4. Discussion

The AztC/D system provides an ideal system to study the mechanisms of zinc binding and direct, protein-protein zinc transfer. Here we have systematically evaluated the role of several structural features of AztC and AztD in these processes. We will discuss the impact of each feature individually.

### 4.1. The AztD N-terminal Motif (NTM)

Some AztD homologues contain motifs rich in His/Asp/Glu residues at either termini or occasionally within the interior of the sequence. For example, the other structurally characterized AztD from *Citrobacter koseri* contains the C-terminal sequence EHHDHEAHHHDDHAH (residues 407–421), which was also absent in that structure due to a lack of electron density [26]. Despite the fact that the termini are situated close to the zinc transfer site in AztD, the NTM appears to play no role in zinc transfer to AztC. However, zinc binding affinity is somewhat altered, with a loss of the third binding site and an apparent loss of negative cooperativity between sites 1 and 2. It is worth noting that an interaction was observed between sites 1 and 2 of adjacent protomers in the crystal structure of *C. koseri* AztD. The fact that we have never observed AztD dimerization in solution initially suggested that this was simply a crystal packing artifact. However, a weak, transient interaction would explain how these sites might communicate and how deletion of the NTM might eliminate that communication.

### 4.2. The AztC Z-Loop

In the holo structure of AztC, the Z-loop is closed over the zinc site with the carbonyl oxygen of Val 224 a distance of 3.6 Å from the zinc ion. While too far to be considered a coordinate bond, this still likely represents an electrostatic interaction. In the apo AztC structure, the loop is pulled away from the zinc site in one chain and absent in the other due to a lack of electron density. This suggests that the Z-loop is highly flexible in apo AztC and that its position is stabilized in the holo form by the interaction between Val 224 and zinc.

The Z-loop is unusual in cluster A-I SBP structures. To our knowledge, it has only been observed in the putative manganese transporter TroA [36,37] and the zinc/laminin binding proteins Lmb [38] and Lbp [39]. In TroA, the zinc-Val interaction is conserved at 3.7–3.9 Å while in Lmb and Lbp the interaction is between the carbonyl oxygen atom of an Ile residue and zinc at 3.4–3.7 Å. Unlike AztC, the apo form of TroA does not show any significant displacement of the Z-Loop relative to the holo form [40]. Although no apo structure exists for Lmb or Lbp, a recent molecular dynamics simulation of both Lmb and TroA indicated significant flexibility of the Z-Loop in the apo form and displacement away from the zinc binding site upon zinc loss [41].

Deletion of the Z-Loop in AztC causes a roughly 6-fold increase in the dissociation rate of zinc relative to WT, suggesting that closure of this loop over the zinc slows its dissociation. The rate of transfer from AztD also increased. The loop is flexible in the apo form and may sample both open and closed conformations on a rapid time scale. The closed form would not be able to interact productively with AztD. Thus, forcing AztC into the open form by deleting this loop would increase the rate of transfer. A direct measurement of dynamics of holo and apo AztC by NMR would be of interest, and these experiments are currently underway in our laboratory.

The presence of a Z-Loop would seem likely to confer an advantage by preventing the premature dissociation of zinc from the SBP. However, most organisms are able to survive zinc limitation with cluster A-I SBPs lacking a Z-Loop, making its physiological function and relevance uncertain. It may also function as a recognition element, allowing specific interaction with the membrane permease. We are currently working to generate Z-Loop deletion mutations in vivo to address this unresolved question.

### 4.3. H138/204A AztC

Our initial intent was to generate a mutant AztC that was able to interact with AztD but not bind zinc. Thus, it could act as a competitive inhibitor of transfer to WT and allow us to estimate a binding affinity between the two proteins. The simplest means to that end seemed to be removing the ligands at the zinc binding site. While we failed to achieve this outcome, the results from this mutant were surprising and may provide some insight into zinc binding and transfer.

Somewhat surprisingly, H138/204A AztC still bound zinc with high affinity, at the limit of the MF-2 assay. However, the off-rate for zinc was very rapid, suggesting that k_on_ for zinc must be near the diffusion limit (~10^8^ M^−1^ × s^−1^) to maintain the observed high affinity. It seems reasonable to assume this is true of WT as well, which would require a Kd for zinc in the mid to low picomolar range (~10^−11^ M). The affinity of most SBPs for zinc is reported in the low nM range [16,42,43,44,45]. However, some of these are only upper limit estimates due to limitations of the binding assays, as is the case for the MF-2 assay. Even using isothermal titration calorimetry (ITC), it can be challenging to accurately measure extremely high binding affinities. Thus, it is possible in many cases, and likely in the case of AztC, that the affinities of some cluster A-I SBPs for zinc are underestimated.

Certainly, the most puzzling result from this work is the observation that inclusion of H138/204A AztC into the WT transfer reaction significantly enhances the rate of transfer. While we can offer no definitive explanation at this time, it would seem to suggest a more complex binding equilibrium between AztC and AztD than previously imagined. It could be that AztC can bind at more than one site on AztD, and that binding to the remote site enhances the rate of transfer. Similarly, some self-association of AztC could speed the process. Evaluation of these possibilities will require interaction assays that are independent of zinc transfer. Labeling AztC and AztD proteins with fluorophores capable of Förster resonance energy transfer (FRET) would seem an attractive strategy. Alternatively, NMR may prove useful in this capacity as well. In any case, continued study of AztC/AztD is likely to provide additional mechanistic insights into zinc transfer between proteins.

## 5. Conclusions

Here we show the role of certain structural features of AztC and AztD in zinc binding and transfer. The NTM of AztD appears to have no role in zinc transfer to AztC but does impact zinc binding. The Z-Loop of AztC functions to lock down over the zinc binding site, decreasing the rate of zinc dissociation. It also appears to inhibit the formation of productive interactions with AztD for transfer. Finally, a double mutant of zinc ligands (H138/204A) of AztC is able to significantly increase the rate of zinc transfer from AztD to WT AztC through an as-yet unknown mechanism. Taken together, this work validates certain aspects of a previously determined interaction model [26] while providing new and interesting avenues of investigation into this unusual system.

## Figures and Tables

**Table 1:** Zinc binding affinity and stoichiometry of WT and mutant AztD.

Protein	Site	K_d_ ± S.D. (nM)	k_off_ ± S.D. (min^−1^)	k_T_ (M^−1^ × s^−1^)
WT AztD [23]	1	0.7 ± 0.3		
	2	54 ± 8		
	3	340 ± 110		
ΔS1 AztD [26]	2	1.3 ± 0.7		
	3	248 ± 81		
ΔS2 AztD [26]	1	0.1 *		
	3	158 ± 35		
ΔNTM AztD	1	0.1 *		1.50 × 10^−3^
	2	0.2 ± 0.1		
WT AztC [24]	1	0.3 ± 0.1	1.2 ± 0.1 × 10^−3^	1.33 × 10^−3^ [26]; 1.55 × 10^−3^ (this work)
ΔD-Loop AztC [25]	1	0.2 ± 0.1	0.9 ± 0.1 × 10^−3^	nd
ΔZ-Loop AztC [25]	1	0.2 *	7.0 ± 0.1 × 10^−3^	2.92 × 10^−3^
H138/204A AztC [24]	1	0.1 *	623.4 ± 0.5 × 10^−3^	

* Uncertainties for this value could not be estimated as the Kd appears to be below the detection limit of this assay. The data was fitted with the indicated value.

**Table 2:** X-ray diffraction data collection, processing and refinement statistics.

	Holo ΔZ-Loop AztC
Data collection	
Space group	P2_1_
Unit cell parameters	
a, b, c (Å) α, β, γ (°)	62.3, 105.0, 64.3 90.0, 110.8, 90.0
Resolution range (Å)	46.6–2.26
Number of reflections (measured/unique)	133,590/36,075
R_merge_	0.04 (0.59)
I/σI	18.5 (2.3)
Completeness (%)	99.5 (99.9)
Redundancy	3.7 (3.7)
Refinement Statistics	
Resolution (Å)	39.5–2.26
R_work_/R_free_	17.4/20.8
Number of atoms	
Protein	3,840
Zinc	2
Water	57
Other	0
R.m.s. deviations	
Bond lengths (Å)	0.009
Bond angles (°)	1.20
Ramachandran Statistics	
Allowed	99.5%
Outliers	0.5%
Average B-factor (Å^2^)	58.0
